# A Three-Item Oral Function Impairment Score Is Associated with White Matter, but Not Gray Matter, Volume Ratio in Older Adults: A Cross-Sectional Study

**DOI:** 10.3390/jcm15218252

**Published:** 2026-10-23

**Authors:** Akihiro Matsumura, Yoshihiro Tamura, Norihiko Narita, Tomoh Matsumiya, Kosei Kubota, Kaito Yanagidate, Yudai Yamaki, Shotaro Mori, Keita Watanabe, Tomohiro Shintaku, Sera Kasai, Kana Saito, Shingo Kakeda, Tatsuya Mikami, Koichi Murashita, Shigeyuki Nakaji, Wataru Kobayashi

**Affiliations:** 1 Department of Oral and Maxillofacial Surgery, Hirosaki University Graduate School of Medicine, Hirosaki 036-8562, Japan; 2 Department of Vascular and Inflammatory Medicine, Biomedical Research Center, Hirosaki University Graduate School of Medicine, Hirosaki 036-8562, Japan; 3 Department of Radiology, Kyoto Prefectural University of Medicine, Kyoto 602-8566, Japan; 4 Department of Radiology, Hirosaki University Graduate School of Medicine, Hirosaki 036-8562, Japan; 5 Department of Social Medicine, Hirosaki University Graduate School of Medicine, Hirosaki 036-8562, Japan; 6 COI Research Initiatives Organization, Hirosaki University Graduate School of Medicine, Hirosaki 036-8562, Japan

**Keywords:** oral function impairment, white matter, gray matter, brain volume, magnetic resonance imaging, community-dwelling older adults, cross-sectional study

## Abstract

**Background/Objectives**: Declining oral function is associated with adverse health outcomes in older adults; however, its relationship with underlying brain structure remains unclear. We examined whether a three-item oral function impairment score (OFIS), based on the number of present teeth, tongue pressure, and oral diadochokinesis (ODK), was associated with white matter (WM) and gray matter (GM) volume ratios in community-dwelling older adults. **Methods**: This cross-sectional study used data from the 2017 Hirosaki Dementia cohort. The primary analysis included 912 participants after excluding those with missing data or a history of neurological conditions; a sensitivity analysis included participants with neurological conditions (*n* = 947). One point was assigned for <20 present teeth, tongue pressure <30 kPa, and reduced ODK, yielding an OFIS ranging from 0 to 3, with higher scores indicating impairment across a greater number of oral function domains. WM and GM volume ratios were calculated as tissue volume divided by total intracranial volume. Associations with OFIS were evaluated using multivariable linear regression adjusted for demographic, lifestyle, and vascular/metabolic factors. **Results**: A higher OFIS was associated with a lower WM volume ratio after multivariable adjustment (B = −0.282; 95% CI, −0.402 to −0.161; *p* < 0.001). No significant association was observed between the OFIS and GM volume ratio after adjustment (B = 0.066; 95% CI, −0.064 to 0.196; *p* = 0.319). The association with WM remained consistent in the sensitivity analysis. In separate covariate-adjusted secondary models, each OFIS component was associated with the WM volume ratio. **Conclusions**: In this cross-sectional study, greater impairment across the three assessed oral function domains was associated with a lower cerebral WM volume ratio, but not GM volume ratio, in community-dwelling older adults.

## 1. Introduction

Declining oral function in older age is an important health concern because it can affect eating, communication, and social participation. In recent years, oral hypofunction and oral frailty, which capture deterioration across multiple domains of oral function, have received increasing attention [1,2]. These conditions are associated with adverse outcomes, including physical frailty, sarcopenia, mild cognitive decline, malnutrition, functional impairment, and mortality [3,4,5], and may therefore provide useful insight into health status and vulnerability in older adults.

Maintaining cognitive function is essential for preserving independence in older adults. Oral health indicators, including tooth count, periodontal status, and masticatory function, have been associated with dementia and cognitive domains such as memory, attention, and executive function [6]. Systematic reviews and meta-analyses of longitudinal studies have shown that poor periodontal health is associated with cognitive decline (odds ratio, 1.23; 95% confidence interval [CI], 1.05–1.44) and dementia (hazard ratio, 1.21; 95% CI, 1.07–1.38) [7]. More recent analyses accounting for cognitive function as a time-varying confounder have similarly identified an association between poor oral health and dementia [8]. Because deterioration in oral status and cognitive decline may be bidirectionally related, examining their relationships with brain structure may help clarify this complex association.

Most previous studies have focused on cognitive test performance or dementia incidence, whereas relatively few have directly examined the relationship between oral function and brain structure. Cognitive test performance can be influenced by cognitive reserve and practice effects from repeated testing [9,10], while magnetic resonance imaging (MRI)-based structural indices provide a quantitative assessment of brain structural changes [11]. Brain volume is widely used as a marker of age-related structural change [12,13] and can identify neurodegeneration before or during the early stages of dementia [14]. White matter (WM) primarily comprises fibers connecting brain regions, whereas gray matter (GM) predominantly contains neuronal cell bodies. WM is particularly susceptible to aging and vascular changes and has been associated with cognitive and physical decline [15,16,17]. Distinguishing WM from GM may therefore provide insight into the structural correlates of oral hypofunction.

Neuroimaging studies have reported associations between tooth loss or overall oral status and brain volume or WM lesions [18,19,20], and a recent study examined a composite oral frailty measure in relation to brain MRI indices [21]. However, previous work has focused largely on regional brain volumes or WM lesions, and few studies have evaluated whole-brain structural indices such as WM and GM volume ratios. Moreover, the relationship between whole-brain volume indices and a composite oral function assessment incorporating the number of present teeth, tongue pressure, and oral diadochokinesis (ODK) remains unclear.

Therefore, this study aimed to examine the cross-sectional associations of a three-item oral function impairment score (OFIS), comprising the number of present teeth, tongue pressure, and ODK, with WM and GM volume ratios in community-dwelling adults aged ≥65 years within the examination year.

## 2. Materials and Methods

### 2.1. Study Design and Participants

This cross-sectional study used data from the 2017 Hirosaki Dementia cohort (Iki-Iki study), a community-based cohort conducted as part of the Japan Prospective Studies Collaboration for Aging and Dementia (JPSC-AD). The cohort includes men and women aged ≥65 years residing in Hirosaki City, Aomori Prefecture, Japan, and was established to elucidate risk factors and develop preventive strategies for cerebrovascular and cardiovascular diseases and dementia [22].

**Figure 1:**
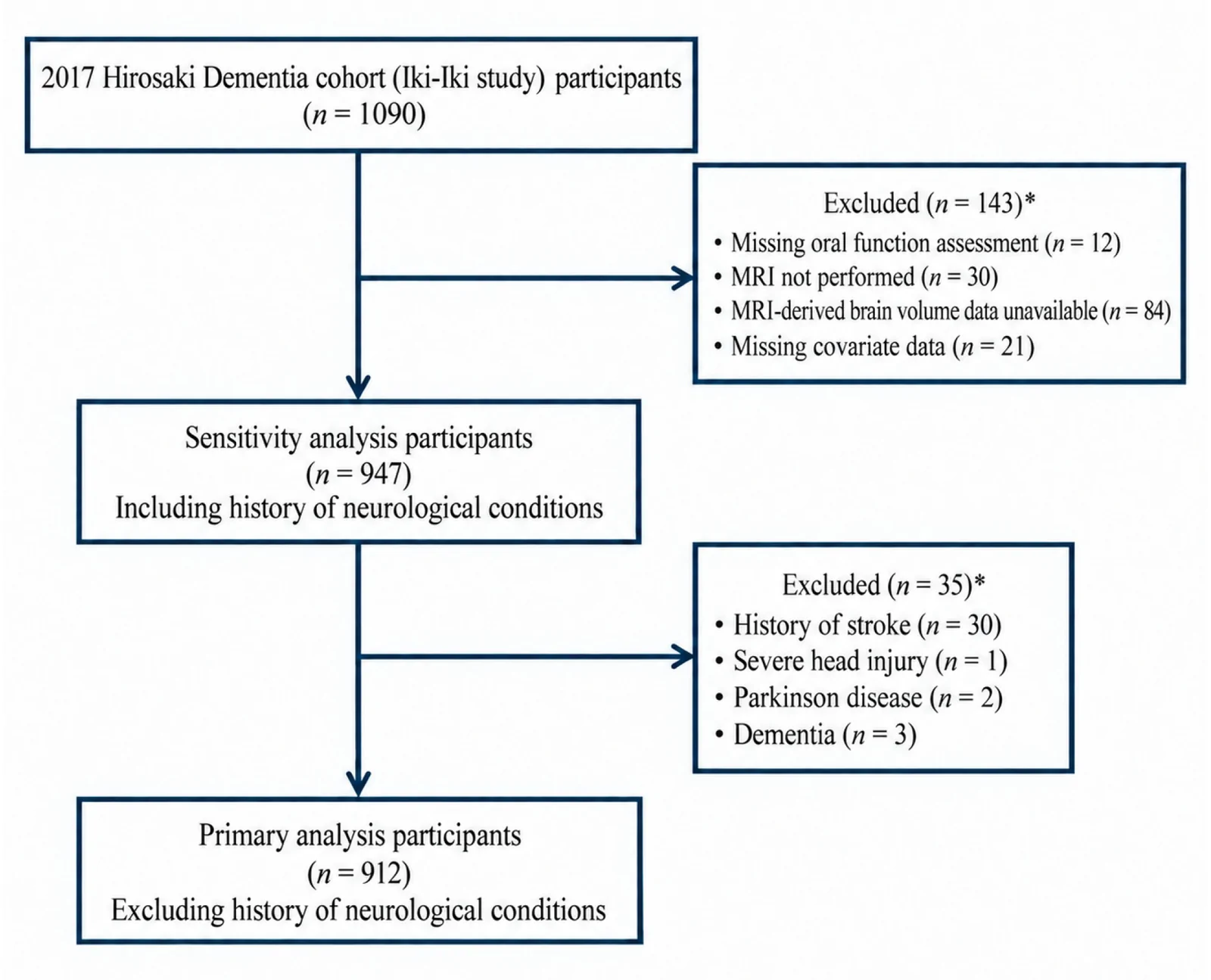
Flowchart of participant selection for the primary and sensitivity analyses. Of the 1090 participants in the 2017 Hirosaki Dementia cohort (Iki-Iki study), 947 with complete oral function, brain magnetic resonance imaging (MRI), and covariate data were included in the sensitivity analysis. After excluding participants with a history of neurological conditions, 912 were included in the primary analysis. * Exclusion categories were not mutually exclusive. MRI-derived brain volume data were unavailable when T1-weighted images were unavailable or when image distortions, such as metal or excessive motion artifacts, precluded accurate measurement of brain volume.

Among the 1090 participants who took part in the 2017 examination and were aged ≥65 years within the study year, those with missing oral function, brain MRI, or covariate data were excluded, leaving 947 participants for the sensitivity analysis. Of these, 912 participants without a history of stroke, severe head injury, Parkinson’s disease, or dementia were included in the primary analysis (Figure 1). These neurological conditions were excluded from the primary analysis because central nervous system impairment can affect both oral function and brain volume through motor or cognitive decline [23,24,25,26]. A complete-case approach was used. Because this study analyzed data from an existing cohort, no a priori sample size calculation was performed; the sample size was determined by the number of eligible participants with complete data from the 2017 examination.

### 2.2. Oral Function Assessment

Among the seven oral hypofunction assessment items proposed by the Japanese Society of Gerodontology, three domains were measured in the 2017 cohort examination and were therefore available for the present analysis: the number of present teeth, tongue pressure, and tongue–lip motor function [1]. The remaining four domains were not assessed in the 2017 examination. Accordingly, the three-item score used in this study does not represent the full seven-domain clinical assessment or diagnosis of oral hypofunction. These three available domains were included because each has been individually associated with frailty [27] and because our group previously applied this three-item combination in a separate cohort [28]. All examinations were performed by dentists according to a standardized protocol.

The number of present teeth was defined as the number of remaining teeth, including third molars and excluding residual roots, and was used as a surrogate marker of occlusal force; <20 teeth were defined as having decreased occlusal force [1]. Tongue pressure was assessed as maximum tongue pressure using a tongue pressure measurement device (TPM-02E; JMS Co., Ltd., Tokyo, Japan), with the maximum of three measurements used for analysis [29]. Maximum tongue pressure <30 kPa was defined as reduced tongue pressure [1]. Tongue–lip motor function was assessed using an ODK measuring device (TKK-3351; Takei Scientific Instruments, Niigata, Japan): the participants repeated the syllables /pa/, /ta/, and /ka/ as quickly as possible for 5 s each, and the number of repetitions per second was calculated [30]. A rate < 6 repetitions/s for any of the three syllables was defined as reduced tongue–lip motor function [1].

One point was assigned for each component that met the criterion for impairment (number of present teeth, tongue pressure, and ODK), yielding the three-item OFIS ranging from 0 to 3, with higher scores indicating impairment across a greater number of the assessed oral function domains.

### 2.3. Brain MRI Acquisition and Image Processing

Brain MRI was performed using a 3.0-tesla MRI scanner (Signa EXCITE; GE Healthcare, Milwaukee, WI, USA). T1-weighted images were acquired in the steady state using a three-dimensional fast-spoiled gradient recalled sequence with the following parameters: repetition time, 10 ms; echo time, 4.1 ms; inversion time, 700 ms; flip angle, 10°; field-of-view, 24 cm; section thickness, 1.2 mm; and resolution, 1.0 × 1.0 × 1.2 mm. Image processing was performed using the Computational Anatomy Toolbox (CAT12, version 12.8.2, revision r2170; Structural Brain Mapping Group, Jena University Hospital, Jena, Germany) implemented in Statistical Parametric Mapping 12 (SPM12, revision r7771; Wellcome Trust Centre for Neuroimaging, London, UK) [31]. T1-weighted images were segmented into GM, WM, and cerebrospinal fluid (CSF) using the default tissue-segmentation settings in CAT12. Spatial normalization was performed using Diffeomorphic Anatomical Registration Through Exponentiated Lie Algebra (DARTEL) [32], and the normalized images were resampled to an isotropic voxel size of 1 mm.

Global GM, WM, and CSF volumes (mL) were obtained from the subject-specific XML reports automatically generated by CAT12 from native-space tissue segmentation [33]. Total intracranial volume (TIV) was defined as the sum of WM, GM, and CSF volumes. WM and GM volume ratios were calculated by dividing the respective tissue volume by TIV, thereby accounting for interindividual differences in intracranial volume. These global volumetric measures were derived from native-space tissue segmentation rather than from spatially normalized or smoothed images.

Participants were excluded from volumetric analysis when T1-weighted images were unavailable or when image distortions, such as metal or excessive motion artifacts, precluded accurate measurement of brain volume. No automated CAT12 quality-score cutoff was used. For these reasons, brain volume data were unavailable for 84 of the 1060 participants who underwent MRI (Figure 1).

### 2.4. Covariates

A standardized self-administered questionnaire used in the JPSC-AD protocol [22] was distributed to the participants in advance, and their responses were confirmed and collected on the day of the examination, followed by individual interviews. The assessed covariates included age, sex, educational attainment (compulsory education/high school/higher education), medical history (hypertension, dyslipidemia, diabetes mellitus), smoking amount (pack-years), alcohol consumption history (current/former/never), and exercise habits. The presence of a regular dental clinic was assessed as a variable in the exploratory analysis. Height and weight were measured on the day of examination, and the body mass index (BMI) was calculated as weight in kilograms divided by height in meters squared (kg/m^2^). Hypertension, dyslipidemia, and diabetes mellitus were included as covariates because they are representative vascular and metabolic risk factors associated with structural brain changes in older adults [34,35].

### 2.5. Statistical Analyses

Participants were categorized into four groups according to OFIS for between-group comparisons. Continuous variables are presented as mean ± standard deviation and were compared using one-way analysis of variance; Welch’s test was used when the assumption of homogeneity of variance was violated. Categorical variables are presented as *n* (%) and were compared using the chi-square test or Fisher’s exact test, as appropriate.

To assess potential selection bias associated with the complete-case analysis, available characteristics were compared between participants included in the complete-case dataset (*n* = 947) and those excluded because of missing data (*n* = 143) using independent-samples t tests (Welch’s test when appropriate) or chi-square tests, based on available data for each variable.

Among participants in the primary analysis, associations of WM and GM volume ratios with each variable were first examined using univariable linear regression. Multivariable linear regression models were then fitted with WM or GM volume ratios as the dependent variable and OFIS as the primary explanatory variable, adjusting for age, sex, educational attainment, smoking amount, alcohol consumption history, exercise habits, BMI, hypertension, dyslipidemia, and diabetes mellitus. OFIS was entered as a single ordinal variable ranging from 0 to 3. To assess the appropriateness of treating OFIS as an ordinal variable, OFIS was additionally modeled as a categorical variable with OFIS 0 as the reference category, and linear, quadratic, and cubic trends were evaluated using polynomial contrasts. Potential nonlinearity in the association between age and WM or GM volume ratio was examined by adding a quadratic term for age centered at 70 years to the multivariable model. Sex, educational attainment, and alcohol consumption history were dummy-coded. For graphical presentation, OFIS was treated as a categorical variable in a general linear model to estimate adjusted marginal means and 95% CIs for WM and GM volume ratios using the same covariates.

For the sensitivity analysis, the primary multivariable model was repeated in the 947 participants, including those with a history of neurological conditions. For an additional sensitivity analysis assessing robustness to the method of intracranial-volume normalization, absolute WM and GM volumes were calculated from the corresponding tissue volume ratios and TIV. Multivariable linear regression models were fitted with absolute WM or GM volume as the dependent variable, with additional adjustment for TIV; all other covariates were identical to those in the primary model. Secondary multivariable analyses among participants in the primary analysis evaluated the individual OFIS components: number of present teeth and tongue pressure as continuous variables and reduced ODK as a binary variable. In an exploratory analysis, univariable and multivariable linear regression models were used to examine having a regular dental clinic as the primary explanatory variable.

Regression results are reported as unstandardized regression coefficients (B), 95% CIs, standardized regression coefficients (β), and *p* values. Multicollinearity was assessed using the variance inflation factor (VIF), with VIF < 10 considered acceptable. Analyses were performed using IBM SPSS Statistics version 31 (IBM Corp., Armonk, NY, USA). All tests were two-sided, and *p* < 0.05 was considered statistically significant. The associations of OFIS with WM and GM volume ratios were considered the primary analyses. No formal adjustment for multiple comparisons was applied. *p* values from the secondary and exploratory analyses were considered nominal and interpreted cautiously.

## 3. Results

### 3.1. Participant Characteristics

The primary analysis included 912 participants (OFIS 0/1/2/3: *n* = 191/346/264/111; Figure 1). Age increased with higher OFIS, whereas the number of present teeth, tongue pressure, and ODK rates for /pa/, /ta/, and /ka/ decreased (all *p* < 0.001; Table 1). Among brain volume indices, the WM volume ratio decreased with increasing OFIS (*p* < 0.001), and the GM volume ratio differed across OFIS groups (*p* = 0.020); TIV did not differ significantly. Similar patterns were observed in the sensitivity-analysis population (Table S1). Compared with the included participants, excluded participants had fewer present teeth (17.9 ± 9.3 vs. 19.7 ± 8.1; *p* = 0.027) and a higher prevalence of neurological conditions (8.4% vs. 3.7%; *p* = 0.010). Other available characteristics did not differ significantly between the groups (Table S2).

### 3.2. Association Between OFISs and WM and GM Volume Ratios

In univariable linear regression, higher OFIS was associated with lower WM (B = −0.609; 95% CI, −0.735 to −0.484; *p* < 0.001) and GM (B = −0.233; 95% CI, −0.385 to −0.080; *p* = 0.003) volume ratios (Table S3).

After multivariable adjustment, higher OFIS remained independently associated with a lower WM volume ratio (B = −0.282; 95% CI, −0.402 to −0.161; *p* < 0.001; Table 2). In contrast, the association with the GM volume ratio was no longer significant (B = 0.066; 95% CI, −0.064 to 0.196; *p* = 0.319). Among the covariates, age, sex, and hypertension were significantly associated with the WM volume ratio, whereas age, sex, and educational attainment were significantly associated with the GM volume ratio. Additional analyses supported the ordinal specification of OFIS: categorical modeling showed progressively lower WM volume ratios with increasing OFIS, and polynomial contrasts demonstrated a significant linear trend (*p* < 0.001) without significant quadratic or cubic components (Table S4). No corresponding trend was observed for GM volume ratio. Addition of a quadratic age term did not materially alter the OFIS estimates.

### 3.3. Adjusted Estimated Marginal Means and Sensitivity Analysis

**Figure 2:**
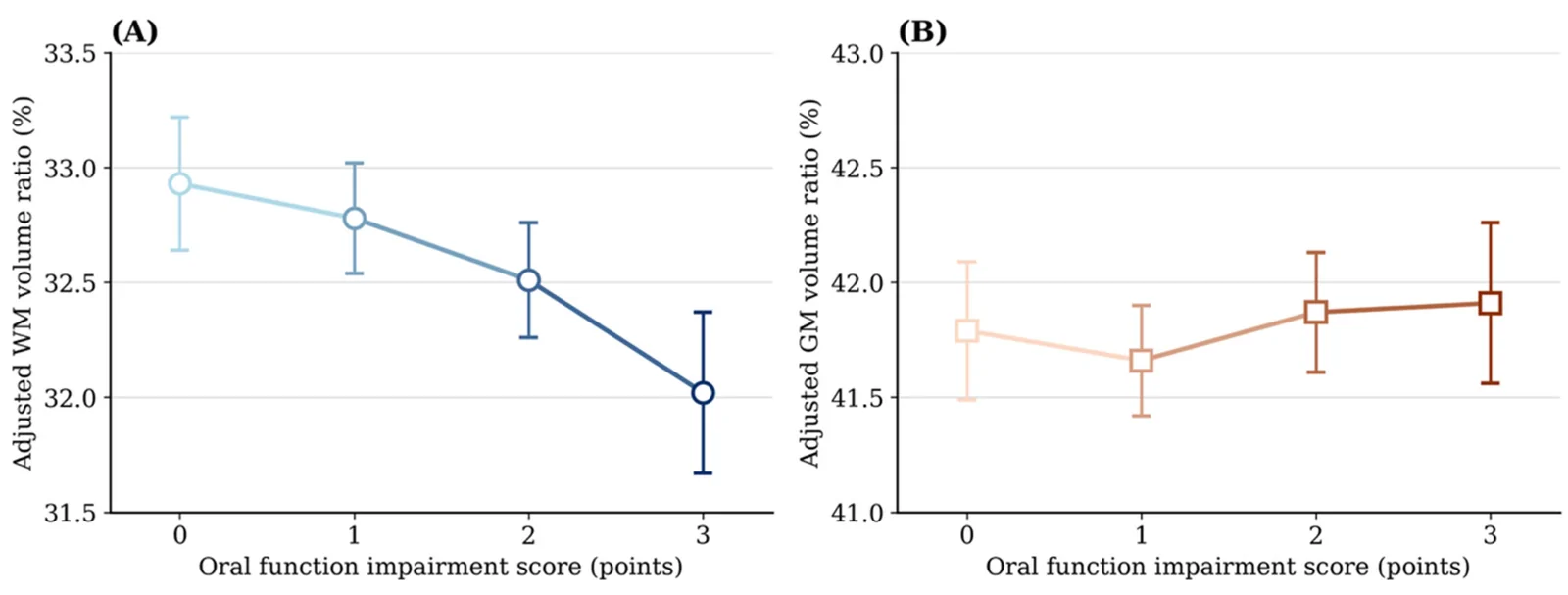
Adjusted estimated marginal means of white and gray matter volume ratios according to oral function impairment score (OFIS). (**A**) White matter (WM) volume ratio and (**B**) gray matter (GM) volume ratio. Marker and line color darken with increasing OFIS (0–3) to visually indicate the severity gradient. The points represent the adjusted estimated marginal means, and the error bars represent the 95% confidence intervals (CIs). The adjusted estimated marginal means were calculated using a general linear model, treating the OFIS as a categorical variable. Each model was adjusted for age, sex, educational attainment, smoking amount (pack-years), alcohol consumption history, exercise habits, body mass index (BMI), hypertension, dyslipidemia, and diabetes mellitus. One point each was assigned for <20 present teeth, maximum tongue pressure < 30 kPa, and reduced ODK (a rate < 6 repetitions/s for any of the /pa/, /ta/, or /ka/ syllables), yielding an OFIS of 0–3. OFIS, oral function impairment score; ODK, oral diadochokinesis.

Adjusted marginal mean WM volume ratios were 32.93%, 32.77%, 32.51%, and 32.02% for OFIS values of 0, 1, 2, and 3, respectively, showing a progressive decrease with increasing OFIS (Figure 2A). The GM volume ratio showed no clear monotonic pattern across OFIS categories (Figure 2B).

In the sensitivity analysis including participants with neurological conditions, higher OFIS remained associated with a lower WM volume ratio (B = −0.292; 95% CI, −0.411 to −0.173; *p* < 0.001), whereas the association with the GM volume ratio remained nonsignificant (B = 0.058; 95% CI, −0.070 to 0.185; *p* = 0.375; Table S5). Figure S1 shows that the negative association with WM was consistent across the univariable, adjusted primary, and adjusted sensitivity analyses. By contrast, the univariable association with GM was attenuated after multivariable adjustment and remained nonsignificant in the sensitivity analysis. In the additional sensitivity analysis using absolute tissue volumes with adjustment for TIV, higher OFIS remained associated with a lower absolute WM volume (B = −3.836 mL; 95% CI, −5.530 to −2.142; *p* < 0.001), whereas no significant association was observed with absolute GM volume (B = 0.610 mL; 95% CI, −1.099 to 2.320; *p* = 0.484; Table S6). These findings were consistent with the primary analyses using WM and GM volume ratios.

### 3.4. Secondary Analysis

In secondary analyses of individual OFIS components, separate multivariable models adjusted for the same covariates as the primary analysis showed positive associations for the number of present teeth (B = 0.014; 95% CI, 0.0002–0.028; *p* = 0.047) and tongue pressure (B = 0.021; 95% CI, 0.005–0.037; *p* = 0.010) with the WM volume ratio, whereas reduced ODK was negatively associated with the WM volume ratio (B = −0.429; 95% CI, −0.647 to −0.211; *p* < 0.001; Table 3). None of the individual oral function measures was significantly associated with the GM volume ratio.

### 3.5. Association Between Having a Regular Dental Clinic and Brain Volume Ratios

Having a regular dental clinic was not significantly associated with the WM volume ratio in the univariable analysis (B = 0.304; 95% CI, −0.002 to 0.610; *p* = 0.051), Model 1 (B = 0.252; 95% CI, −0.014 to 0.519; *p* = 0.063), or Model 2, which additionally adjusted for OFIS (B = 0.242; 95% CI, −0.022 to 0.506; *p* = 0.072; Table S7). No significant association with the GM volume ratio was observed in any model.

## 4. Discussion

In this study of community-dwelling older adults, higher OFIS, integrating the number of present teeth, tongue pressure, and ODK, was associated with a lower WM volume ratio. This association persisted after adjustment for demographic, lifestyle, and vascular/metabolic factors and remained consistent in the sensitivity analysis. By contrast, the univariable association between the OFIS and GM volume ratio was attenuated after multivariable adjustment.

Previous neuroimaging studies have linked tooth loss to WM changes and silent brain infarction [36] and periodontal disease-related indices to WM hyperintensities and impaired WM microstructure [37]. Our findings are consistent with this broader evidence linking poor oral health to WM structure. However, the WM volume ratio used here is a relative volumetric measure and does not directly assess WM lesions or microstructure. Accordingly, our results complement, rather than establish a common pathological mechanism for, previous observations relating oral health to WM-related MRI indices.

Several mechanisms could potentially contribute to the differing association patterns observed for WM and GM. First, reduced oral motor activity may be accompanied by diminished orofacial sensorimotor stimulation. Because neuronal activity promotes oligodendrogenesis and adaptive myelination, reduced oral sensorimotor activity could plausibly influence WM [38]. Second, mastication increases cerebral blood flow and oxygenation, suggesting that reduced masticatory activity may diminish this hemodynamic stimulation [39]. Third, impaired oral processing has been associated with lower energy and nutrient intake in older adults [40], potentially affecting WM through metabolic and vascular pathways. Given the susceptibility of cerebral WM to chronic hypoperfusion and small-vessel injury [41], these pathways may partly account for the observed association. However, none of these mechanisms was directly assessed in this study, and these interpretations remain speculative.

The findings may also be considered in the context of brain reserve, which refers to structural resources, such as brain volume and neuronal number, contributing to resilience against aging and pathological change [42]. WM supports connectivity across distributed brain regions and is particularly susceptible to vascular and age-related changes [15]. Thus, a lower WM volume ratio among participants with a higher OFIS may be compatible with greater structural vulnerability. However, brain reserve was not directly measured, and WM volume ratio should not be considered a surrogate for brain reserve; this interpretation is therefore hypothesis-generating.

Although OFIS was associated with the GM volume ratio in univariable analysis, this association was not evident after multivariable adjustment. Age, sex, and educational attainment were strongly associated with the GM volume ratio, suggesting that confounding by these background factors may have contributed to the attenuation. Educational attainment has also been associated with regional and age-related GM volume loss [43,44]. Our findings therefore do not exclude the possibility of a relationship between oral function and GM structure; rather, they indicate that an independent association with whole-brain GM volume ratio was not evident after accounting for measured covariates.

A notable feature of this study is the use of a three-item composite score integrating the number of present teeth, tongue pressure, and ODK. These measures correspond to three domains included in the oral hypofunction framework proposed by the Japanese Society of Gerodontology; however, the OFIS used in the present study does not represent the full seven-domain clinical assessment of oral hypofunction. In separate secondary analyses, greater numbers of present teeth and higher tongue pressure, modeled as continuous variables, were positively associated with the WM volume ratio, whereas reduced ODK, modeled as a binary variable, was negatively associated. Because these analyses were not adjusted for multiplicity, the findings should be interpreted cautiously, particularly the borderline association observed for the number of present teeth. Nevertheless, the direction of association was consistent across the three components, with better oral function corresponding to a higher WM volume ratio. Multidomain assessment of oral function may capture overall health status more comprehensively than any individual measure alone [45].

Tooth loss has been associated with subsequent cognitive decline and dementia [46], while reduced tongue pressure and ODK have been associated with cognitive decline and mild cognitive impairment [47,48]. Although cognitive function and incident dementia were not evaluated as outcomes in the present analysis, the association between the composite OFIS and WM volume ratio provides complementary structural evidence relevant to the relationship between oral and cognitive health. Importantly, the WM volume ratio is not a surrogate for cognitive decline, and whether OFIS predicts future cognitive outcomes requires evaluation in longitudinal studies incorporating cognitive assessment.

In the exploratory analysis, having a regular dental clinic was not independently associated with the WM volume ratio. Nevertheless, dental visiting behavior has been associated with subsequent dementia incidence in a longitudinal study [49], and may warrant further evaluation in prospective studies.

The clinical relevance of these findings should be considered in the context of their cross-sectional nature. Readily assessed oral function measures were associated with WM volume ratio, but the present findings do not establish OFIS as a screening tool or biomarker for cerebral structural change. Rather, they identify a potential relationship between multidomain oral function impairment and relative cerebral WM volume that warrants further longitudinal investigation. In the adjusted categorical estimates, the WM volume ratio was approximately 0.91 percentage points lower in participants with an OFIS of 3 than in those with a score of 0 (32.02% vs. 32.93%), corresponding to an approximately 2.8% relative difference. Previous studies have reported that lower WM fraction was associated with poorer cognitive performance [50], whereas lower WM volume was associated with poorer physical function [51]. However, these measures are not directly comparable with the whole-brain WM volume ratio normalized to TIV examined in the present study, and no established threshold defines a clinically meaningful difference for this specific measure. Because cognitive and functional outcomes were not evaluated in the present analysis, the clinical significance of the observed difference remains uncertain. Accordingly, the magnitude of the association should be interpreted as a structural imaging finding rather than as evidence of clinically meaningful impairment.

This study has several limitations. First, its cross-sectional design precludes determination of temporality or causality between the OFIS and WM volume ratio. Reverse causation is possible because brain structural aging or subclinical neurological changes may themselves contribute to poorer oral motor function or oral health. Thus, the observed association may reflect a bidirectional relationship rather than a unidirectional effect of oral function on WM volume. Second, WM and GM volume ratios are relative whole-brain structural indices and do not directly characterize WM lesions, WM microstructure, or regional brain volumes. They may also be influenced by changes in other intracranial compartments, including CSF volume, limiting inference regarding the underlying pathology. Third, the OFIS incorporated only three of the seven domains used in the clinical assessment of oral hypofunction because the remaining four domains were not measured in the 2017 cohort examination. Therefore, the OFIS should not be interpreted as equivalent to the full clinical assessment or diagnosis of oral hypofunction. In addition, the number of present teeth did not fully account for denture use or prosthetic status and may therefore not accurately reflect functional occlusal status. Fourth, complete-case analysis may have introduced selection bias because excluded participants had fewer present teeth and a higher prevalence of neurological conditions. In particular, brain volume data were unavailable for 84 participants (7.9% of those who underwent MRI) because T1-weighted images were unavailable or image distortions, due to metal or excessive motion artifacts, precluded accurate measurement of brain volume. These participants may have differed from those included in brain pathology or general health, and the direction and magnitude of any resulting bias could not be determined. Fifth, because participants voluntarily attended a community health examination, they may have been healthier or more health-conscious than the general older population, potentially limiting the generalizability of the findings. Sixth, residual confounding by unmeasured factors, including dietary and nutritional factors, socioeconomic circumstances, cognitive reserve, and subclinical vascular disease, cannot be excluded despite adjustment for multiple covariates. Moreover, some vascular and metabolic conditions included as covariates could potentially lie on pathways linking oral function to brain structure; adjustment for these factors may therefore have attenuated the observed association. Because their causal roles cannot be determined in this cross-sectional study, possible overadjustment should be considered when interpreting the adjusted estimates. Seventh, cognitive and functional outcomes were not evaluated in the present analysis; therefore, the observed WM volume ratio differences cannot be directly linked to cognitive or functional impairment. Finally, analyses of individual OFIS components and dental visiting behavior were secondary or exploratory, were not adjusted for multiplicity, and should be interpreted cautiously.

## 5. Conclusions

In this cross-sectional study, a higher three-item OFIS was independently associated with a lower WM volume ratio, whereas no independent association was observed with the GM volume ratio. These findings support further investigation of the relationship between multidomain oral function impairment and relative cerebral WM volume in older adults. Longitudinal studies incorporating cognitive outcomes are needed to clarify the temporal and clinical significance of this association.

## Figures and Tables

**Table 1:** Characteristics of the participants based on OFIS (primary analysis participants).

	Overall (*n* = 912)	OFIS 0 (*n* = 191)	OFIS 1 (*n* = 346)	OFIS 2 (*n* = 264)	OFIS 3 (*n* = 111)	*p* Value
Demographic characteristics						
Age (years)	70.0 ± 4.3	67.7 ± 3.3	69.6 ± 4.0	71.1 ± 4.3	72.6 ± 4.2	* <0.001
Sex						0.911
Male	340 (37.3)	75 (39.3)	125 (36.1)	98 (37.1)	42 (37.8)	
Female	572 (62.7)	116 (60.7)	221 (63.9)	166 (62.9)	69 (62.2)	
Educational attainment						0.772
Compulsory education	173 (19.0)	36 (18.8)	59 (17.1)	54 (20.5)	24 (21.6)	
High school	466 (51.1)	95 (49.7)	187 (54.0)	127 (48.1)	57 (51.4)	
Higher education	273 (29.9)	60 (31.4)	100 (28.9)	83 (31.4)	30 (27.0)	
Lifestyle factors						
Smoking amount (pack-years)	9.1 ± 18.8	9.2 ± 17.4	10.2 ± 20.0	8.8 ± 20.1	6.4 ± 13.6	0.318
Alcohol consumption history						0.173
Never	433 (47.5)	94 (49.2)	157 (45.4)	130 (49.2)	52 (46.8)	
Current	420 (46.1)	91 (47.6)	169 (48.8)	111 (42.0)	49 (44.1)	
Former	59 (6.5)	6 (3.1)	20 (5.8)	23 (8.7)	10 (9.0)	
Exercise habits	619 (67.9)	131 (68.6)	242 (69.9)	174 (65.9)	72 (64.9)	0.648
Physical and metabolic factors						
BMI (kg/m^2^)	22.8 ± 3.1	22.8 ± 2.8	22.9 ± 3.2	22.8 ± 3.2	22.5 ± 2.9	0.686
Hypertension	400 (43.9)	83 (43.5)	167 (48.3)	108 (40.9)	42 (37.8)	0.151
Dyslipidemia	390 (42.8)	77 (40.3)	164 (47.4)	103 (39.0)	46 (41.4)	0.165
Diabetes mellitus	143 (15.7)	30 (15.7)	61 (17.6)	38 (14.4)	14 (12.6)	0.549
Dental care-related factors						
Having a regular dental clinic	724 (79.4)	159 (83.2)	269 (77.7)	210 (79.5)	86 (77.5)	0.465
Oral function-related indices						
Number of present teeth	19.6 ± 8.1	25.3 ± 2.6	21.5 ± 7.3	16.8 ± 8.3	10.8 ± 6.4	* <0.001
Tongue pressure (kPa)	31.5 ± 6.9	36.0 ± 4.4	32.4 ± 6.2	30.0 ± 7.0	24.3 ± 5.2	* <0.001
ODK /pa/ (repetitions/s)	6.5 ± 0.6	6.8 ± 0.5	6.6 ± 0.6	6.2 ± 0.6	6.1 ± 0.6	* <0.001
ODK /ta/ (repetitions/s)	6.4 ± 0.7	6.8 ± 0.5	6.5 ± 0.6	6.0 ± 0.6	5.8 ± 0.6	<0.001
ODK /ka/ (repetitions/s)	5.9 ± 0.6	6.4 ± 0.4	6.0 ± 0.6	5.6 ± 0.5	5.4 ± 0.5	* <0.001
Brain volume-related indices						
Total intracranial volume (mL)	1371.70 ± 123.08	1375.17 ± 122.00	1374.83 ± 121.23	1371.39 ± 122.25	1356.72 ± 132.81	0.569
White matter volume ratio (%)	32.6 ± 1.9	33.4 ± 1.7	32.8 ± 1.7	32.3 ± 2.0	31.4 ± 2.0	<0.001
Gray matter volume ratio (%)	42.2 ± 2.2	42.6 ± 2.0	42.2 ± 2.2	42.1 ± 2.3	41.8 ± 2.5	* 0.02

Values are represented as mean ± standard deviation or *n* (%). One point was assigned for each of <20 present teeth, maximum tongue pressure < 30 kPa, and reduced ODK (a rate < 6 repetitions/s for any of the /pa/, /ta/, or /ka/ syllables), yielding an OFIS of 0–3. Between-group comparisons for continuous variables were performed using one-way analysis of variance. * Welch’s test was applied when the assumption of homogeneity of variance was not met. Categorical variables were compared using the chi-square test. OFIS, oral function impairment score; ODK, oral diadochokinesis.

**Table 2:** Multivariable linear regression analysis for white and gray matter volume ratios (primary analysis participants).

Explanatory Variable	White Matter Volume Ratio			Gray Matter Volume Ratio		
	B (95% CI)	β	*p* Value	B (95% CI)	β	*p* Value
Primary explanatory variable						
OFIS (points)	−0.282 (−0.402, −0.161)	−0.139	<0.001	0.066 (−0.064, 0.196)	0.028	0.319
Demographic characteristics						
Age (years)	−0.204 (−0.231, −0.178)	−0.459	<0.001	−0.181 (−0.209, −0.153)	−0.35	<0.001
Male (vs. female)	−0.223 (−0.442, −0.003)	−0.057	0.047	−2.258 (−2.494, −2.022)	−0.494	<0.001
Educational attainment (reference: compulsory education)						
High school	−0.070 (−0.358, 0.218)	−0.018	0.632	0.427 (0.117, 0.736)	0.096	0.007
Higher education	0.042 (−0.274, 0.358)	0.01	0.794	0.408 (0.068, 0.748)	0.084	0.019
Lifestyle factors						
Smoking amount (pack-years)	−0.002 (−0.008, 0.004)	−0.024	0.437	−0.0003 (−0.007, 0.006)	−0.003	0.927
Alcohol consumption history (reference: never)						
Current	−0.099 (−0.329, 0.132)	−0.026	0.401	−0.114 (−0.362, 0.134)	−0.026	0.367
Former	−0.201 (−0.654, 0.252)	−0.026	0.384	−0.372 (−0.860, 0.115)	−0.041	0.134
Exercise habits	−0.108 (−0.338, 0.121)	−0.027	0.354	−0.171 (−0.479, 0.136)	−0.037	0.275
Physical and metabolic factors						
BMI (kg/m^2^)	0.003 (−0.033, 0.039)	0.005	0.858	−0.023 (−0.062, 0.016)	−0.032	0.245
Hypertension	−0.229 (−0.454, −0.004)	−0.06	0.046	0.216 (−0.026, 0.458)	0.048	0.081
Dyslipidemia	−0.070 (−0.287, 0.148)	−0.018	0.530	0.096 (−0.138, 0.331)	0.022	0.419
Diabetes mellitus	0.295 (−0.002, 0.592)	0.056	0.051	−0.046 (−0.365, 0.273)	−0.008	0.927
Adjusted R^2^	0.282			0.384		

B, unstandardized regression coefficient; β, standardized regression coefficient; 95% CI, 95% confidence interval. The multivariable model was adjusted for age, sex, educational attainment, smoking amount (pack-years), alcohol consumption history, exercise habits, BMI, hypertension, dyslipidemia, and diabetes mellitus. The OFIS was treated as an ordinal scale ranging from 0 to 3, and the regression coefficient represented the change per 1-point increase in the OFIS. Educational attainment and alcohol consumption history were entered as dummy-coded variables. Reference categories are shown in the table. OFIS, oral function impairment score; BMI, body mass index.

**Table 3:** Multivariable linear regression analysis of white and gray matter volume ratios according to the OFIS component (primary analysis participants).

Model	Primary Explanatory Variable	White Matter Volume Ratio			Gray Matter Volume Ratio		
		B (95% CI)	β	*p* Value	B (95% CI)	β	*p* Value
Model 1	Number of present teeth	0.014 (0.0002, 0.028)	0.059	0.047	0.003 (−0.011, 0.018)	0.013	0.641
Model 2	Tongue pressure (kPa)	0.021 (0.005, 0.037)	0.077	0.010	−0.006 (−0.023, 0.011)	−0.018	0.505
Model 3	Reduced ODK (yes/no)	−0.429 (−0.647, −0.211)	−0.113	<0.001	0.115 (−0.118, 0.349)	0.026	0.333

B, unstandardized regression coefficient; β, standardized regression coefficient; 95% CI, 95% confidence interval. For unstandardized coefficients (B), estimates for the number of present teeth and tongue pressure represent the change per 1 tooth and per 1 kPa, respectively. Reduced ODK was coded as yes versus no, with no as the reference category. Only the coefficients for the primary explanatory variables are shown. Each model included one OFIS component as the primary variable. β values are not directly comparable across components because reduced ODK was binary, whereas present teeth and tongue pressure were continuous. The multivariable model was adjusted for age, sex, educational attainment, smoking amount (pack-years), alcohol consumption history, exercise habits, BMI, hypertension, dyslipidemia, and diabetes mellitus. Reduced ODK was defined as a rate of <6 repetitions/s for any of the /pa/, /ta/, or /ka/ syllables. Educational attainment and alcohol consumption history were entered as dummy-coded variables. Reference categories are shown in the table. OFIS, oral function impairment score; ODK, oral diadochokinesis; BMI, body mass index.

## Data Availability

The data used in this study were derived from the Hirosaki Iki-Iki study, which is part of the Japan Prospective Studies Collaboration for Aging and Dementia. These data are not publicly available because of privacy restrictions under the Japanese Act on the Protection of Personal Information and terms of informed consent obtained from participants. Data access may be considered upon reasonable request from the corresponding author, subject to institutional ethics board approval, and a data sharing agreement.

## Supplementary Materials

The following supporting information can be downloaded at https://www.mdpi.com/article/10.3390/jcm15218252/s1. Figure S1: Forest plot of the associations between OFIS and white and gray matter volume ratios in the univariable, adjusted primary, and adjusted sensitivity analyses; Table S1: Characteristics of participants according to OFIS (sensitivity analysis participants); Table S2: Comparison of characteristics between participants included in the complete-case dataset and those excluded because of missing data; Table S3: Univariable linear regression analysis for white and gray matter volume ratios (primary analysis participants); Table S4: Additional analyses assessing the linearity assumptions for the three-item oral function impairment score and age (primary analysis participants); Table S5: Multivariable linear regression analysis for white and gray matter volume ratios (sensitivity analysis participants); Table S6: Multivariable linear regression analysis for absolute white and gray matter volumes with adjustment for total intracranial volume (additional sensitivity analysis; primary analysis participants); Table S7: Association between having a regular dental clinic and white and gray matter volume ratios: univariable and multivariable linear regression analysis (primary analysis participants).

## Abbreviations

The following abbreviations are used in this manuscript:

OFIS	oral function impairment score
ODK	oral diadochokinesis
WM	white matter
GM	gray matter
MRI	magnetic resonance imaging
JPSC-AD	Japan Prospective Studies Collaboration for Aging and Dementia
CSF	cerebrospinal fluid
TIV	total intracranial volume
BMI	body mass index
CIs	confidence intervals
VIF	variance inflation factor
