# Supplementary material for: A Three-Item Oral Function Impairment Score Is Associated with White Matter, but Not Gray Matter, Volume Ratio in Older Adults: A Cross-Sectional Study
Source: J Clin Med. 2026 Oct 23;15(21):8252. doi: 10.3390/jcm15218252 (PMC13763676; doi:10.3390/jcm15218252)
Supplement: Supplementary file 1 [file jcm-15-08252-s001.zip › Supplementary Figure S1.pdf]

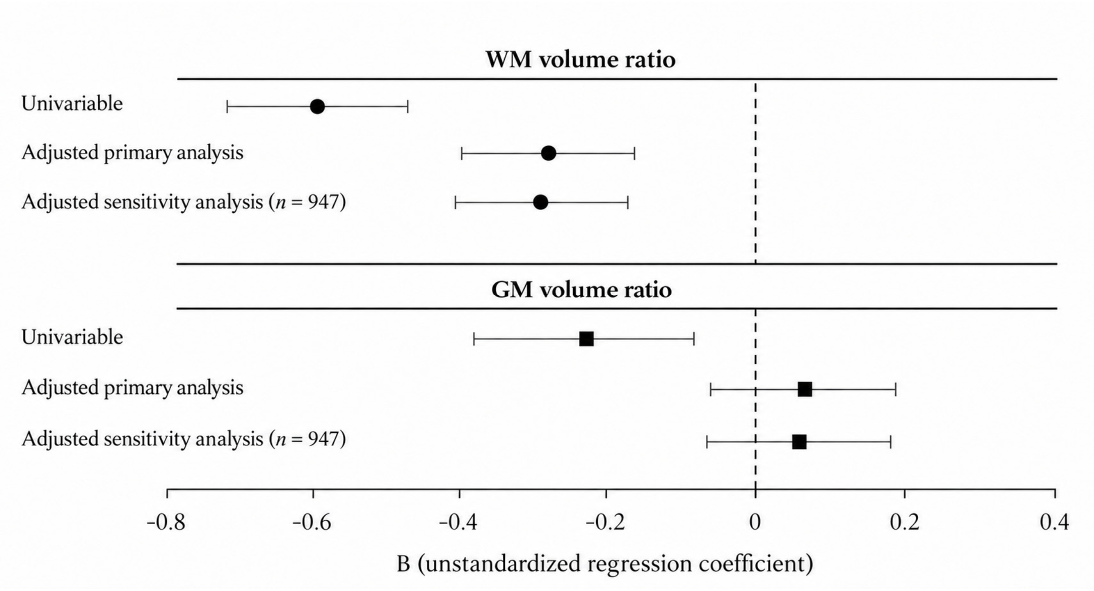

Supplementary Figure S1. Forest plot of the associations between OFIS and white and gray matter volume ratios in the univariable, adjusted primary, and adjusted sensitivity analyses.

The points represent unstandardized regression coefficients (B), and the error bars represent the 95% confidence intervals (CIs). Each estimate represents the regression coefficient for a 1-point increase in the OFIS. The multivariable analysis was adjusted for age, sex, educational attainment, smoking amount (pack-years), alcohol consumption history, exercise habits, body mass index (BMI), hypertension, dyslipidemia, and diabetes mellitus. Sensitivity analysis was performed on a population that included participants with a history of neurological conditions. The dashed line indicates B = 0. OFIS, oral function impairment score
