# Supplementary material for: A Three-Item Oral Function Impairment Score Is Associated with White Matter, but Not Gray Matter, Volume Ratio in Older Adults: A Cross-Sectional Study
Source: J Clin Med. 2026 Oct 23;15(21):8252. doi: 10.3390/jcm15218252 (PMC13763676; doi:10.3390/jcm15218252)
Supplement: Supplementary file 1 [file jcm-15-08252-s001.zip › Supplementary Tables S1-S7.pdf]

Supplementary Table S1. Characteristics of participants according to OFIS score (sensitivity analysis participants)

	**Overall (*n* = 947)**	**OFIS 0 (*n* = 202)**	**OFIS 1 (*n* = 360)**	**OFIS 2 (*n* = 272)**	**OFIS 3 (*n* = 113)**	**P value**
Demographic characteristics						
Age (years)	70.0±4.3	67.7±3.3	69.5±4.0	71.0±4.3	72.7±4.2	*<0.001
Sex						0.868
Male	348 (36.7)	78 (38.6)	127 (35.3)	100 (36.8)	43 (38.1)	
Female	599 (63.3)	124 (61.4)	233 (64.7)	172 (63.2)	70 (61.9)	
Educational attainment						0.803
Compulsory education	183 (19.3)	41 (20.3)	62 (17.2)	56 (20.6)	24 (21.2)	
High school	483 (51.0)	100 (49.5)	193 (53.6)	131 (48.2)	59 (52.2)	
Higher education	281 (29.7)	61 (30.2)	105 (29.2)	85 (31.3)	30 (26.5)	
Lifestyle factors						
Smoking amount (pack-years)	9.3±19.0	9.5±17.6	10.1±20.0	9.2±20.6	6.5±13.7	0.376
Alcohol consumption history						0.328
Never	447 (47.2)	98 (48.5)	164 (45.6)	132 (48.5)	53 (46.9)	
Current	435 (45.9)	95 (47.0)	175 (48.6)	115 (42.3)	50 (44.2)	
Former	65 (6.9)	9 (4.5)	21 (5.8)	25 (9.2)	10 (8.8)	
Exercise habits	644 (68.0)	140 (69.3)	251 (69.7)	180 (66.2)	73 (64.6)	0.645
Physical and metabolic factors						
BMI (kg/m^2)	22.8±3.0	22.8±2.8	22.9±3.1	22.8±3.2	22.5±2.9	0.705
Hypertension	426 (45.0)	91 (45.0)	176 (48.9)	115 (42.3)	44 (38.9)	0.196

Dyslipidemia	408 (43.1)	82 (40.6)	172 (47.8)	107 (39.3)	47 (41.6)	0.144
Diabetes mellitus	148 (15.6)	32 (15.8)	64 (17.8)	38 (14.0)	14 (12.4)	0.434
History of neurological conditions						
Stroke	30 (3.2)	10 (5.0)	12 (3.3)	8 (2.9)	0 (0)	0.118
Parkinson disease	2 (0.2)	1 (0.5)	1 (0.3)	0 (0)	0 (0)	**0.781
Severe head injury	1 (0.1)	0 (0)	0 (0)	0 (0)	1 (0.9)	**0.119
Dementia	3 (0.3)	0 (0)	2 (0.6)	0 (0)	1 (0.9)	**0.253
Dental care-related factors						
Having a regular dental clinic	751 (79.3)	169 (83.7)	279 (77.5)	216 (79.4)	87 (77.0)	0.331
Oral function-related indices						
Number of present teeth	19.7±8.1	25.3±2.6	21.5±7.3	17.0±8.3	10.8±6.4	*<0.001
Tongue pressure (kPa)	31.5±6.9	36.0±4.5	32.4±6.2	30.0±7.1	24.3±5.1	*<0.001
ODK /pa/ (repetitions/s)	6.5±0.7	6.9±0.5	6.6±0.6	6.2±0.6	6.1±0.6	*<0.001
ODK /ta/ (repetitions/s)	6.4±0.7	6.8±0.5	6.5±0.6	6.0±0.6	5.8±0.6	<0.001
ODK /ka/ (repetitions/s)	5.9±0.6	6.4±0.4	6.0±0.6	5.6±0.5	5.4±0.5	*<0.001
Brain volume-related indices						
Total intracranial volume (mL)	1371.76±123.18	1376.47±122.20	1372.74±121.26	1372.89±122.24	1357.48±133.48	0.603
White matter volume ratio (%)	32.6±1.9	33.3±1.7	32.8±1.8	32.2±1.9	31.4±2.0	<0.001
Gray matter volume ratio (%)	42.2±2.2	42.6±2.0	42.2±2.2	42.1±2.3	41.8±2.5	*0.017

Values are reported as means ± standard deviations or *n* (%). One point was assigned for each of < 20 present teeth, maximum tongue pressure < 30 kPa, and reduced ODK (a rate < 6 repetitions/s for any of the /pa/, /ta/, or /ka/ syllables), yielding an OFIS of 0–3. Between-group comparisons for continuous variables were performed using one-way analysis of variance; *Welch's test was applied when the assumption of homogeneity of variance was not met. Categorical variables were compared using the chi-square test or **Fisher's exact test. OFIS, oral function impairment score; ODK, oral diadochokinesis.

Supplementary Table S2. Comparison of characteristics between participants included in the complete-case dataset and those excluded because of missing data

Variable	Included participants (*n* = 947)	Excluded participants (*n* = 143)		P value
	Value	Available *n*	Value	
Demographic characteristics				
Age (years)	70.0 ± 4.3	143	70.1 ± 4.3	0.656
Sex		143		0.473
Male	348 (36.7)		57 (39.9)	
Female	599 (63.3)		86 (60.1)	
Educational attainment		142		0.605
Compulsory education	183 (19.3)		32 (22.5)	
High school	483 (51.0)		72 (50.7)	
Higher education	281 (29.7)		38 (26.8)	
Lifestyle factors				
Smoking amount (pack-years)	9.3 ± 19.0	133	7.7 ± 15.5	0.360
Alcohol consumption history		142		0.692
Never	447 (47.2)		72 (50.7)	
Current	435 (45.9)		62 (43.7)	
Former	65 (6.9)		8 (5.6)	
Exercise habits	644 (68.0)	143	98 (68.5)	0.900
Physical and metabolic factors				
BMI (kg/m^2)	22.8 ± 3.0	140	23.1 ± 2.8	0.365
Hypertension	426 (45.0)	141	66 (46.8)	0.685
Dyslipidemia	408 (43.1)	141	61 (43.3)	0.968

Diabetes mellitus	148 (15.6)	143	20 (14.0)	0.612
Neurological history				
History of neurological conditions	35 (3.7)	143	12 (8.4)	0.010
Oral function-related indices				
Number of present teeth	19.7 ± 8.1	134	17.9 ± 9.3	0.027*
Tongue pressure (kPa)	31.5 ± 6.9	138	31.5 ± 6.4	0.992
ODK /pa/ (repetitions/s)	6.5 ± 0.7	141	6.5 ± 0.8	0.705*
ODK /ta/ (repetitions/s)	6.4 ± 0.7	141	6.3 ± 0.7	0.801
ODK /ka/ (repetitions/s)	5.9 ± 0.6	141	5.9 ± 0.8	0.685*

Values are represented as mean ± standard deviation or *n* (%). Percentages were calculated using the available observations for each variable. Between-group comparisons for continuous variables were performed using independent-samples t tests. *Welch's t test was applied when the assumption of homogeneity of variance was not met. Categorical variables were compared using the chi-square test. Neurological conditions included stroke, severe head injury, Parkinson’s disease, and dementia. BMI, body mass index; ODK, oral diadochokinesis.

Supplementary Table S3. Univariable linear regression analysis for white and gray matter volume ratios (primary analysis participants)

Explanatory variable	White matter volume ratio		Gray matter volume ratio	
	B (95% CI)	P value	B (95% CI)	P value
Primary explanatory variable				
OFIS (points)	-0.609 (-0.735, -0.484)	<0.001	-0.233 (-0.385, -0.080)	0.003
Demographic characteristics				
Age (years)	-0.229 (-0.254, -0.205)	<0.001	-0.188 (-0.219, -0.156)	<0.001
Male (vs. female)	-0.280 (-0.536, -0.024)	0.013	-2.358 (-2.613, -2.103)	<0.001
Educational attainment (reference: compulsory education)				
High school	-0.084 (-0.417, 0.250)	0.623	0.520 (0.134, 0.905)	0.008
Higher education	0.115 (-0.248, 0.479)	0.533	0.370 (-0.051, 0.790)	0.085
Lifestyle factors				
Smoking amount (pack-years)	-0.003 (-0.009, 0.004)	0.419	-0.0005 (-0.008, 0.007)	0.904
Alcohol consumption history (reference: never)				
Current	-0.146 (-0.402, 0.110)	0.264	-0.179 (-0.476, 0.118)	0.238
Former	-0.505 (-1.024, 0.013)	0.056	-0.338 (-0.940, 0.264)	0.271
Exercise habits	-0.111 (-0.376, 0.155)	0.414	-0.171 (-0.479, 0.136)	0.275
Physical and metabolic factors				
BMI (kg/m^2)	0.001 (-0.039, 0.042)	0.955	-0.021 (-0.068, 0.025)	0.369
Hypertension	-0.191 (-0.441, 0.058)	0.133	0.246 (-0.043, 0.535)	0.096
Dyslipidemia	-0.091 (-0.341, 0.160)	0.478	0.045 (-0.246, 0.335)	0.763
Diabetes mellitus	0.253 (-0.087, 0.594)	0.145	0.016 (-0.379, 0.412)	0.935

B indicates unstandardized regression coefficient; 95% CI, 95% confidence interval. OFIS was treated as an ordinal scale ranging from 0 to 3, and the regression coefficient represents the change per 1-point increase in OFIS. Educational attainment and alcohol consumption history were entered as dummy-coded variables. Reference categories are shown in the table. OFIS, oral function impairment score; BMI, body mass index.

Supplementary Table S4. Additional analyses assessing the linearity assumptions for the three-item oral function impairment score and age (primary analysis participants)

A. Categorical analysis of OFIS				
OFIS category	White matter volume ratio		Gray matter volume ratio	
	B (95% CI)	P value	B (95% CI)	P value
0	Reference	—	Reference	—
1	-0.159 (-0.449, 0.131)	0.283	-0.130 (-0.442, 0.182)	0.414
2	-0.419 (-0.734, -0.104)	0.009	0.084 (-0.256, 0.423)	0.629
3	-0.908 (-1.310, -0.507)	<0.001	0.128 (-0.304, 0.560)	0.562
**B. Polynomial contrasts across OFIS categories**				
**Contrast**	**White matter volume ratio**		**Gray matter volume ratio**	
		**P value**		**P value**
Linear		<0.001		0.384
Quadratic		0.159		0.490
Cubic		0.771		0.281
**C. Analysis including a quadratic term for age**				
**Variable**	**White matter volume ratio**		**Gray matter volume ratio**	
	**B (95% CI)**	**P value**	**B (95% CI)**	**P value**
OFIS (per 1-point increase)	-0.281 (-0.402, -0.160)	<0.001	0.062 (-0.068, 0.192)	0.348
Age^2 (centered at 70 years)	0.000 (-0.006, 0.006)	0.950	-0.005 (-0.011, 0.001)	0.115

B indicates unstandardized regression coefficient; 95% CI, 95% confidence interval. OFIS was modeled as a categorical variable in Panel A, with OFIS 0 as the reference category. All models were adjusted for age, sex, educational attainment, smoking amount (pack-years), alcohol consumption history, exercise habits, BMI, hypertension, dyslipidemia, and diabetes mellitus. Polynomial contrasts in Panel B were evaluated using the same covariate adjustment. In Panel C, age was centered at 70 years before calculation of the quadratic term, and the linear age term was included simultaneously in the model. OFIS, oral function impairment score; BMI, body mass index.

Supplementary Table S5. Multivariable linear regression analysis for white and gray matter volume ratios (sensitivity analysis participants)

Explanatory variable	White matter volume ratio			Gray matter volume ratio		
	**B (95% CI)**	**β**	**P value**	**B (95% CI)**	**β**	**P value**
Primary explanatory variable						
OFIS (points)	-0.292 (-0.411, -0.173)	-0.144	<0.001	0.058 (-0.070, 0.185)	0.024	0.375
Demographic characteristics						
Age (years)	-0.200 (-0.226, -0.174)	-0.447	<0.001	-0.182 (-0.210, -0.154)	-0.35	<0.001
Male (vs. female)	-0.239 (-0.456, -0.022)	-0.061	0.031	-2.264 (-2.496, -2.031)	-0.493	<0.001
Educational attainment (reference: compulsory education)						
High school	-0.096 (-0.377, 0.185)	-0.025	0.502	0.373 (0.071, 0.674)	0.084	0.015
Higher education	-0.007 (-0.317, 0.303)	-0.002	0.965	0.365 (0.031, 0.698)	0.075	0.032
Lifestyle factors						
Smoking amount (pack-years)	-0.003 (-0.009, 0.003)	-0.033	0.264	-0.001 (-0.007, 0.005)	-0.009	0.753
Alcohol consumption history (reference: never)						
Current	-0.091 (-0.319, 0.137)	-0.024	0.433	-0.101 (-0.346, 0.143)	-0.023	0.416

Former	-0.231 (-0.667, 0.206)	-0.031	0.300	-0.297 (-0.766, 0.172)	-0.034	0.214
Exercise habits	-0.124 (-0.351, 0.103)	-0.03	0.283	-0.213 (-0.457, 0.030)	-0.045	0.086
Physical and metabolic factors						
BMI (kg/m²)	0.005 (-0.031, 0.040)	0.007	0.802	-0.030 (-0.068, 0.009)	-0.041	0.131
Hypertension	-0.267 (-0.488, -0.045)	-0.07	0.018	0.198 (-0.039, 0.436)	0.045	0.102
Dyslipidemia	-0.063 (-0.277, 0.151)	-0.016	0.564	0.076 (-0.154, 0.305)	0.017	0.518
Diabetes mellitus	0.272 (-0.021, 0.565)	0.052	0.069	-0.022 (-0.336, 0.293)	-0.004	0.892
Adjusted R^2	0.277			0.383		

B indicates unstandardized regression coefficient; β, standardized regression coefficient; 95% CI, 95% confidence interval. Sensitivity analysis participants were defined using complete-case analysis, including participants with a history of neurological conditions. The multivariable model was adjusted for age, sex, educational attainment, smoking amount (pack-years), alcohol consumption history, exercise habits, BMI, hypertension, dyslipidemia, and diabetes mellitus. OFIS was treated as an ordinal scale ranging from 0 to 3, and the regression coefficient represents the change per 1-point increase in OFIS. Educational attainment and alcohol consumption history were entered as dummy-coded variables. Reference categories are shown in the table. OFIS, oral function impairment score; BMI, body mass index.

Supplementary Table S6. Multivariable linear regression analysis for absolute white and gray matter volumes with adjustment for total intracranial volume (additional sensitivity analysis; primary analysis participants)

**Explanatory variable**	**Absolute white matter volume**		**Absolute gray matter volume**	
	**B (95% CI)**	**P value**	**B (95% CI)**	**P value**
OFIS (points)	-3.836 (-5.530, -2.142)	<0.001	0.610 (-1.099, 2.320)	0.484

B indicates unstandardized regression coefficient; 95% CI, 95% confidence interval. Absolute white and gray matter volumes were reconstructed from the corresponding tissue volume ratios and total intracranial volume (TIV). The sensitivity analysis used absolute white or gray matter volume as the dependent variable and additionally adjusted for TIV. The multivariable model was adjusted for age, sex, educational attainment, smoking amount (pack-years), alcohol consumption history, exercise habits, BMI, hypertension, dyslipidemia, diabetes mellitus, and TIV. OFIS was treated as an ordinal scale ranging from 0 to 3, and the regression coefficient represents the change in absolute tissue volume (mL) per 1-point increase in OFIS. Educational attainment and alcohol consumption history were entered as dummy-coded variables. Reference categories are shown in the table. OFIS, oral function impairment score; BMI, body mass index; TIV, total intracranial volume.

Supplementary Table S7. Association between having a regular dental clinic and white and gray matter volume ratios: univariable and multivariable linear regression analysis (primary analysis participants)

**Primary explanatory variable**	**Model**	**White matter volume ratio**		**Gray matter volume ratio**	
		**B (95% CI)**	**P value**	**B (95% CI)**	**P value**
Having a regular dental clinic: yes (vs no)	Univariable analysis	0.304 (-0.002, 0.610)	0.051	-0.104 (-0.460, 0.251)	0.565
	Multivariable analysis (Model 1)	0.252 (-0.014, 0.519)	0.063	0.031 (-0.253, 0.315)	0.831
	Multivariable analysis (Model 2)	0.242 (-0.022, 0.506)	0.072	0.033 (-0.251, 0.317)	0.818

B indicates unstandardized regression coefficient; 95% CI, 95% confidence interval. In univariable analysis, having a regular dental clinic was examined as the primary explanatory variable. Model 1 was adjusted for age, sex, educational attainment, smoking amount (pack-years), alcohol consumption history, exercise habits, BMI, hypertension, dyslipidemia, and diabetes mellitus. Model 2 was additionally adjusted for OFIS in addition to Model 1. OFIS, oral function impairment score; BMI, body mass index.
